# Association between the homeostasis model assessment of insulin resistance and coronary artery calcification: a meta-analysis of observational studies

**DOI:** 10.3389/fendo.2023.1271857

**Published:** 2023-11-27

**Authors:** Longti Li, Huiqin Zhong, Ya Shao, Yu Hua, Xu Zhou, Desheng Luo

**Affiliations:** ^1^ Department of Nursing, TaiHe Hospital, Hubei University of Medicine, Shiyan, China; ^2^ Innovation Centre of Nursing Research, TaiHe Hospital, Hubei University of Medicine, Shiyan, China; ^3^ Health Management Center, TaiHe Hospital, Hubei University of Medicine, Shiyan, China

**Keywords:** insulin resistance, homeostasis model assessment, HOMA-IR, coronary artery calcification, CAC, meta-analysis

## Abstract

**Background:**

Insulin resistance (IR), a risk factor for cardiovascular diseases, has garnered significant attention in scientific research. Several studies have investigated the correlation between IR and coronary artery calcification (CAC), yielding varying results. In light of this, we conducted a systematic review to investigate the association between IR as evaluated by the homeostasis model assessment (HOMA-IR) and CAC.

**Methods:**

A comprehensive search was conducted to identify relevant studies in PubMed, Embase, Scopus, and Web of Science databases. In addition, preprint servers such as Research Square, BioRxiv, and MedRxiv were manually searched. The collected data were analyzed using either fixed or random effects models, depending on the heterogeneity observed among the studies. The assessment of the body of evidence was performed using the GRADE approach to determine its quality.

**Results:**

The current research incorporated 15 studies with 60,649 subjects. The analysis revealed that a higher category of HOMA-IR was associated with a greater prevalence of CAC in comparison to the lowest HOMA-IR category, with an OR of 1.13 (95% CI: 1.06–1.20, I^2^ = 29%, P < 0.001). A similar result was reached when HOMA-IR was analyzed as a continuous variable (OR: 1.27, 95% CI: 1.14–1.41, I^2^ = 54%, P < 0.001). In terms of CAC progression, a pooled analysis of two cohort studies disclosed a significant association between increased HOMA-IR levels and CAC progression, with an OR of 1.44 (95% CI: 1.04–2.01, I^2^ = 21%, P < 0.05). It is important to note that the strength of the evidence was rated as low for the prevalence of CAC and very low for the progression of CAC.

**Conclusion:**

There is evidence to suggest that a relatively high HOMA-IR may be linked with an increased prevalence and progression of CAC.

## Introduction

Insulin resistance (IR) refers to a pathological condition where the capacity of insulin to facilitate the absorption and utilization of glucose is compromised due to diverse factors. Consequently, the body secretes excessive insulin, leading to hyperinsulinemia (1). Studies indicate that IR is independent of conventional risk factors but is closely linked to cardiovascular disease (2), including coronary artery disease and unfavorable cardiovascular events (3–5). One of the key mechanisms by which IR contributes to cardiovascular disease is the promotion of arterial stiffness, impaired vasodilation, and calcification (6, 7). Following the introduction of the homeostasis model assessment of IR (HOMA-IR) by Matthews (8), it has become widely used in clinical research. Numerous studies have demonstrated its excellent correlation with the gold-standard hyperinsulinemic clamp test (9).

Coronary artery calcification (CAC) is an important indicator of subclinical arteriosclerosis (10), leading to reduced vascular compliance and consequently affecting myocardial perfusion. The timely detection of factors associated with CAC in the general population has a favorable impact on the subsequent cardiovascular disease burden (11). Studies have established a strong association between moderate-to-severe CAC and adverse cardiovascular events (12). At the same time, CAC proves to be a dependable instrument for predicting the probability of upcoming cardiovascular occurrences, especially in individuals who show no symptoms (13). The progression from CAC to a severe cardiovascular event often follows a gradual pathophysiologic process, and individuals typically display no apparent signs or symptoms until the cardiovascular event occurs.

Although the relationship between IR and adverse cardiovascular events is well established by research, the relationship between IR and CAC lacks sufficient evidence. Several studies have revealed a strong link between the two (14, 15), whereas others have found no significant correlation (16–18). The computed tomography-based Agatston score (19) is frequently utilized as a non-invasive approach for evaluating CAC. Considering this, based on the Agatston method, the current study performed a meta-analysis to explore the association between IR and CAC.

## Methods

This study was not registered on any platform, and the protocol was not published anywhere. However, we strictly adhere to the Preferred Reporting items for Systematic Reviews and Meta-analyses (20). See **Supplementary File 1**.

### Search strategy

Two independent researchers systematically searched the PubMed, Embase, Scopus, and Web of Science databases. Manual searches were also performed on the Research Square, BioRxiv, and MedRxiv preprint servers to reduce the potential impact of publication bias. Retrieval strategies incorporating Boolean operators were utilized for the following keywords: (1) “insulin resistance” OR “homeostasis model assessment” OR “HOMA” and (2) “coronary artery calcification” OR “coronary calcification” OR “coronary artery calcium” OR “coronary calcium” OR “subclinical coronary atherosclerosis.” A supplementary manual search was conducted through references to relevant literature. The final literature search was updated to 16/09/2023. A detailed search strategy is provided in **Supplementary File 2**. Endnote software was used in the literature screen process.

### Selection of studies

The criteria for inclusion in the literature were as follows: (1) adult participants; (2) reported on the relationship between IR and CAC; (3) assessed IR using the homeostasis model assessment of IR (HOMA-IR) method; (4) evaluated the prevalence or progression of CAC based on the Agatston score; (5) reported effect estimates while accounting for underlying confounding variables. Conference abstracts, case reports, reviews, letters, comments, and expert opinions were excluded. CAC was defined using the definition provided in the original study. The effect estimates were evaluated using risk, hazard, or odds ratio (OR) values.

### Data extraction and quality assessment

Literature retrieval, data extraction, and quality evaluation were performed independently by two researchers (YH and XZ). In case of any discrepancies, a consensus was reached by consulting a third researcher (YS) to resolve the dispute. If vital data for analysis were found to be missing, we would approach the respective paper’s corresponding author to acquire the original data. The following information was extracted: author information, year, country, research type, study population characteristics, sample size, age, gender, disease status, HOMA-IR analysis method, reported outcomes, inclusion and exclusion criteria of subjects, variables adjusted, and outcome definitions. To evaluate the quality of the literature incorporated in this research, we applied the Newcastle–Ottawa Scale (NOS) (21). This scale, which employs a scoring system ranging from 0 to 9, was utilized for assessing the selection of research populations, comparability among cohorts, and outcome measurement.

### Statistical analysis

We utilized pooled ORs to assess the association between HOMA-IR and CAC. One study separately reported ORs for the relationship between HOMA-IR and CAC based on glycated hemoglobin quartiles (22), whereas another reported ORs for different CAC risk levels (23). Therefore, we combined these groupings, calculated ORs, and analyzed them using a random-effects model. We selected the most appropriately adjusted model for confounding factors in multifactor analyses with multiple models and extracted the corresponding estimates.

When HOMA-IR was analyzed as a categorical variable, we extracted the estimates of its highest quartile to the first quartile. When HOMA-IR was presented as a continuous variable, we extracted the value of each unit increase in the variable. To ensure a normal distribution, a log transformation was applied to both the ORs and their corresponding 95% CIs. Heterogeneity was evaluated using the Cochrane Q and I^2^ tests (24). A P-value below 0.1 for the Q-test indicated significant heterogeneity. Heterogeneity was considered low if I^2^ was less than 40%, whereas 40%–60% indicated moderate heterogeneity. In cases where heterogeneity was evident, sources of heterogeneity were sought based on study characteristics and sensitivity analyses. If heterogeneity persisted, a random effects model was employed to combine the information and adjust for different sample sizes, which helped mitigate heterogeneity’s impact (25).

In order to guarantee the dependability of the combined findings, a sensitivity analysis was performed, wherein each study was sequentially excluded, and the corresponding effect on the pooled OR estimates was evaluated (26). Further subgroup analyses were performed to investigate potential differences between integrated ORs with different characteristics, such as demographics and comorbid conditions. In addition, the symmetry of funnel plots was assessed to determine the potential for publication bias. We utilized RevMan (Version 5.1; Cochrane Collaboration, Oxford, UK) and STATA software to conduct this systematic review.

### Certainty of evidence

Two researchers (LL and HZ) utilized the GRADE (Grading of Recommendations, Assessment, Development and Evaluation) approach to assess the quality of evidence. Following the GRADE principle, the initial level of evidence obtained from observational research was deemed as having a low quality. Five factors were taken into account to diminish the credibility of the evidence, which encompassed limitations in the study design, inconsistency, indirectness, imprecision, and the presence of publication bias (27). Additionally, three factors enhance the quality of evidence: large effect sizes, potential confounders, and dose–response relationships (28). If factors increased the evidence quality during the evaluation, it was upgraded to moderate quality. Conversely, if factors diminished the quality of evidence, it was downgraded to very low (29).

## Results

### Literature search

**Figure 1:**
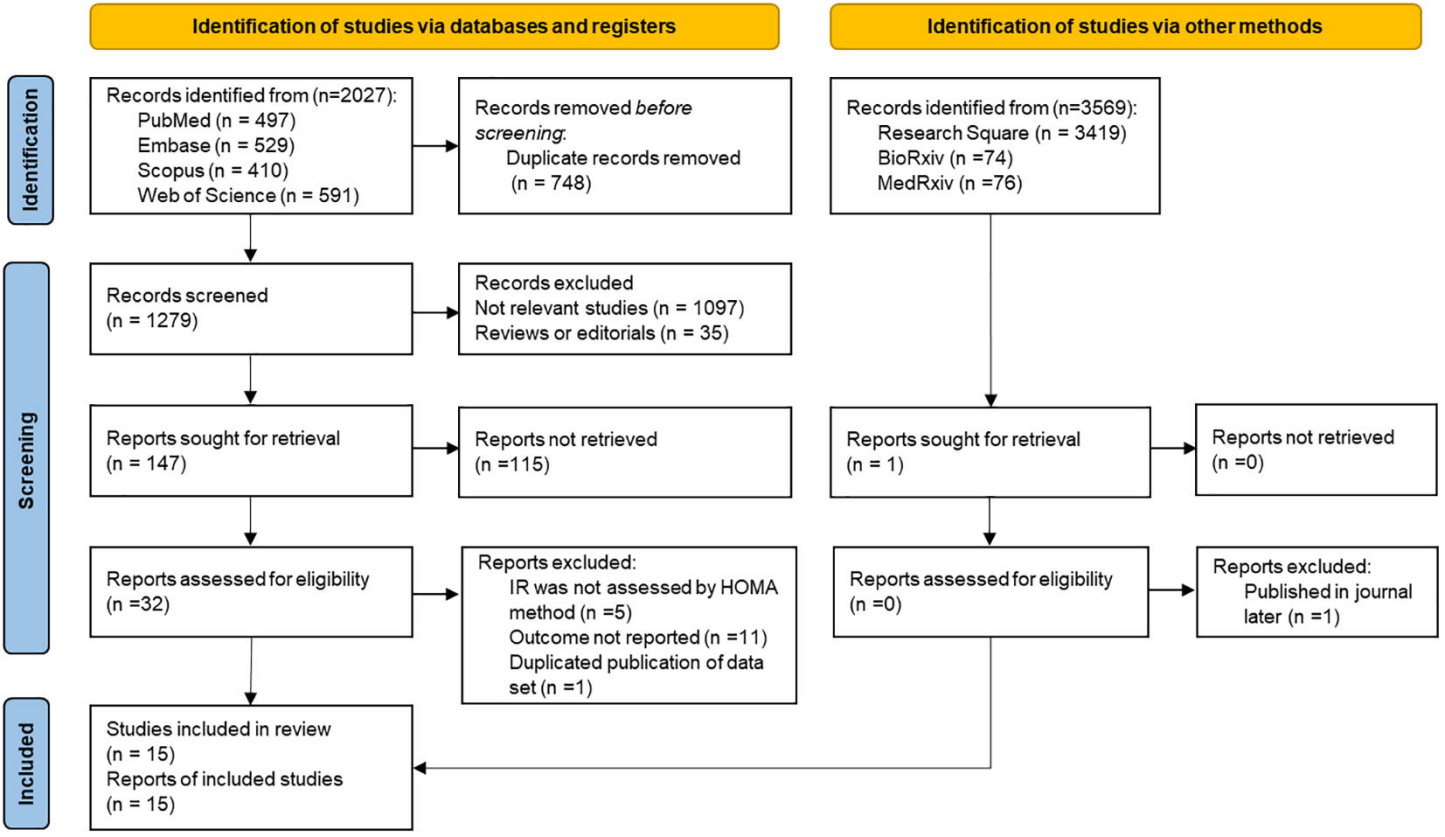
Flowchart of literature selection and screening process.

Following the exclusion of duplicate articles in the initial literature search, a total of 1,279 articles were obtained. However, upon reviewing the titles and abstracts, 1,247 articles were deemed irrelevant and subsequently excluded from further consideration. After a thorough review of 32 relevant studies, it was found that five of them used a non-HOMA-IR method for IR evaluation, and 11 did not report study-related results. One study was excluded to avoid duplication of data sets based on a survey of the same population (30), and we used a more recent study of this group. Ultimately, 15 articles were included in the current research (14, 15, 17, 18, 22, 23, 31–39). The entire process of literature selection is shown in **Figure 1**.

### Study characteristics and quality assessment

**Table 1** presents a comprehensive overview of the selected studies comprising 60,649 subjects from 15 studies. The studies were carried out across multiple countries, comprising the United States (17, 18, 23, 32, 35, 36, 38), South Korea (14, 22, 31, 37, 39), Iran (33), Mexico (34), and Japan (15). Five were cohort studies (14, 15, 18, 31, 38), whereas the remaining 10 were cross-sectional (17, 22, 23, 32–37, 39). The population of the studies comprised community residents or citizens (15, 17, 18, 33–36, 38), participants undergoing health screening (14, 22, 31, 32, 37, 39), and chronic kidney disease patients (23). There were 12 studies that excluded individuals with cardiovascular disease (CVD) (14, 17, 18, 22, 31–37, 39). One study partially excluded individuals with CVD by only excluding those with coronary heart disease (15), whereas two other studies included individuals with CVD in their study population (23, 38).

**Table 1:** Characteristics of the included studies.

Study	Country	Design	Participant	No.	Age	HTN (%)	DM (%)	HOMA-IR analysis	Outcomes reported	Inclusion and exclusion criteria of subjects	Variables adjusted	Definition of the outcomes	NOS score
Bertoni (17)	United States	CS	Communities	5,810	61.7	40.6	0	Q5:Q1	CAC prevalence	Participants without DM and prior CVD were included	Race, metabolic syndrome, HTN medication, HDL-C, triglycerides, pressure, and WC	Agatston score >0	9
Blaha (18)	United States	PC	Communities	5,464	62	NA	NA	Q4:Q1	CAC prevalence	Patients without CVD were included; excluded participants with hypoglycemic therapy	Age, sex, ethnicity, research site, CAC scans years, WC, fasting glucose, low HDL-C, triglycerides, and HTN	Agatston score >0	9
Cho (31)	South Korea	PC	Medical checkup population	1,145	54.2	33.0	13.5	Q4:Q1	CAC progression	Participants who underwent health examinations were included; excluded with a history of CVD or percutaneous coronary intervention or coronary artery bypass surgery, use of statins	Age, sex, systolic blood pressure, LDL-C, HDL-C, smoking, drinking, exercise habits, baseline CAC score, and follow-up interval	Agatston score = 0 at baseline or an increment of 2.5 units between the square root of the baseline and final Agatston scores	8
Echouffo-Tcheugui (32)	United States	CS	Medical checkup population	3,257	54.5	0.4	19.8	Q4:Q1; continuous	CAC prevalence	Participants without CVD (myocardial infarction, coronary artery bypass graft, stroke, heart failure) and with ankle-brachial index <1.40 were included	Age, sex, BMI, smoking, alcohol, ratio of total cholesterol to HDL-C, systolic pressure, use of antihypertensive or statins, and eGFR	Agatston score >0	8
Fakhrzadeh (33)	Iran	CS	Citizens	DM:105; non-DM:103	DM:54.1; non- DM:49.7	70.9	50.5	Continuous	CAC prevalence	Free of CVD, and no abnormal electrocardiogram was included	Age, sex, BMI, WC, LDL-C, HDL-C, HTN, hyperlipidemia	Agatston score >10	7
He (23)	United States	CS	Chronic kidney disease	2,018	NA	86.7	46.6	Continuous	CAC prevalence	Mild-to-moderate CKD was included; with cirrhosis, human immunodeficiency virus infection, polycystic kidney disease, renal cell carcinoma, dialysis or kidney transplant, and taking immunosuppressant drugs or coronary artery revascularization were excluded	Age, gender, race, smoking, previous CVD, HTN, DM, use of lipid-lowering drugs, BMI, WC, and cystatin C	Participants were classified as no (0) moderate (0-100) or high risk (>100) based on Agatston score	8
Jorge-Galarza (34)	Mexico	CS	Communities	1,201	53.6	NA	13.4	Q4:Q1	CAC prevalence	Without a history of CHD or CVD were included; with a history of renal, liver, thyroid, or malignant disease or treatment with corticosteroids were excluded	Age, gender, BMI, smoking, activity, statin use, blood pressure, LDL-C, HDL-C, triglycerides, eGFR, hs C-reactive protein, adiponectin, DM, and visceral adipose tissue.	Agatston score >0	8
Jung (22)	South Korea	RC	Medical checkup population	18,504	40.8	16.3	0	Q4:Q1	CAC prevalence	Participants without DM and CVD were included	Age, SBP, TC, TG, HDL-C, BMI, and smoking	Agatston score >0	9
Ke (38)	United States	PC	Communities	2,777	50.1	32.5	10.3	Q3:Q1	CAC prevalence	Participants are to be 18-30 years at the initial examination and be free of a long-term disease or disability	Age, sex, race, BMI, smoking, creatinine, blood pressure, LDL-C, HbA1c	Agatston score >0	8
Kim (39)	South Korea	CS	Medical checkup population	4,319	NA	0	0	Q4:Q1	CAC prevalence	Participants who underwent health examinations were enrolled; elevated triglyceride, malignancy, inflammatory or infectious disease, history of angina, myocardial infarction or cerebrovascular accidents, HTN, DM, renal disease, taking statins or triglyceride-lowering medications were excluded	Age, sex, systolic blood pressure, BMI, LDL-C, HDL-C, smoking, alcohol, and exercise habits	Agatston score >0	8
Mehta (35)	United States	CS	Communities	DM:611; non-DM:803	DM:60; non-DM:48	NA	0	Continuous	CAC prevalence	Participants without of CVD, elevated creatinine, and the presence of DM were included	Age, gender, medications, HTN, dyslipidemia, alcohol, exercise, C-reactive protein, metabolic syndrome, apolipoprotein B	Agatston score >0	8
Reilly (36)	United States	CS	Communities	840	NA	37.0	0	Continuous	CAC prevalence	Participants without CVD were included; with DM, total cholesterol >300 mg/dL, smoking, or blood pressure >160/100 mmHg were excluded	Age, LDL-C, smoking, exercise, alcohol, race, family history of premature CAD, and medications (aspirin, statins, ACE inhibitors, and hormone replacement therapy)	Participants were classified as mild (11–100), moderate (100–400) or high risk (>400) based on their Agatston score	8
Sung (37)	South Korea	CS	Medical checkup population	10,511	38.9	NA	3.5	Q4:Q1 continuous	CAC prevalence	Patients aged 30–45 years old who participated in health examination were included; a history of CAD were excluded	Age, smoking, HTN, LDL-C, HDL-C, BMI	Agatston score >0	9
Sung (14)	South Korea	PC	Medical checkup population	2,175	42.5	24.0	8.6	Q4:Q1	CAC progression	Patients who participated in health examination were included; a history of cancer and CVD were excluded	Age, sex, year, alcohol, smoking, exercise, education, DM, HTN, medication for lipids or HTN or DM, LDL-C, eGFR, and hs-CRP	Subjects had increased follow-up Agatston scores compared with baseline	9
Yamazoe (15)	Japan	PC	Citizens	796	63.8	50.2	0	Continuous	CAC prevalence; CAC progression	Men aged 40–79 years were selected from the general population; prior CHD, missing data on fasting serum insulin, and triglyceride ≥400 mg/dL were excluded	Age, smoking, alcohol, LDL-C, HTN, dyslipidemia, use of anti-lipid medications, and BMI	CAC prevalence: Agatston score >0; CAC progression: Agatston score = 0 at baseline had a score >0 at follow-up; Agatston score <100 at baseline had an annualized change of ≥10 Agatston units; Agatston score ≥100 at baseline had an annualized percentage change of ≥10%	8

HTN, hypertension; DM, diabetes mellitus; HOMA-IR, homeostasis model assessment of insulin resistance; NOS, Newcastle–Ottawa Scale; CS, cross-sectional; PC, prospective cohort; RC: retrospective cohort; CVD, cardiovascular disease; HDL-C, high-density lipoprotein cholesterol; LDL-C, low-density lipoprotein cholesterol; WC, waist circumference; NA, not available; CAC, coronary artery calcification; BMI, body mass index; eGFR, estimated glomerular filtrating rate; CKD, chronic kidney disease; CHD, coronary heart disease; CAD, coronary artery disease.

The participants involved in the studies exhibited a considerable range in size, with sample sizes ranging from 208 to 18,504. Additionally, the studies displayed diversity in the mean age of the participants, ranging from 38.8 to 63.8 years. In terms of the analysis of HOMA-IR, eight studies used categorical variables (14, 17, 18, 22, 31, 34, 38, 39), whereas five used continuous variables (15, 23, 33, 35, 36) and two used both (32, 37). There were 12 studies that reported CAC prevalence (17, 18, 22, 23, 32–39), two reported CAC progression (14, 31), and one reported CAC prevalence and progression in both (15).

The studies used different criteria to determine the prevalence and progression of CAC, with all studies utilizing the Agatston score methodology for assessment. For the prevalence of CAC, 10 studies used an Agatston score above 0 as the criterion for “judging” (15, 17, 18, 22, 32, 34, 35, 37–39), one had a CAC score more than 10 (33), and two studies classified it into multiple risk classes based on the Agatston score (23, 36). Regarding the progression of CAC, three studies had their criteria for judging the progression of CAC (14, 15, 31). The NOS score for the selected studies ranged from 7 to 9. Details are presented in **Table 1**.

### HOMA-IR and CAC prevalence

**Figure 2:**
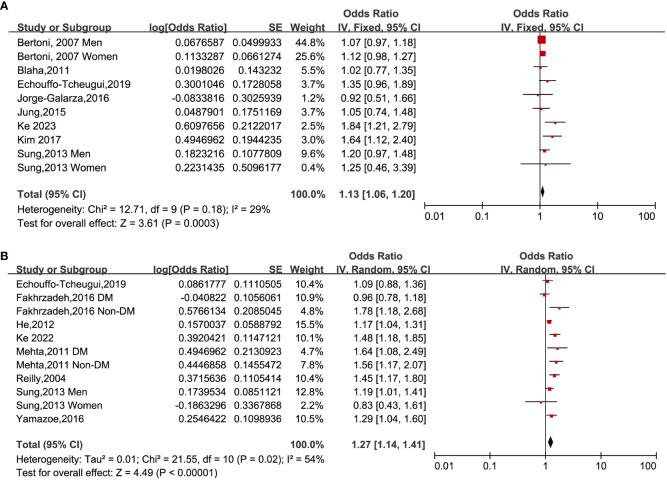
Forest plots of the ORs for CAC prevalence associated with the HOMA-IR. **(A)**: HOMA-IR was estimated as a categorical variable. **(B)**: HOMA-IR was estimated as a continuous variable.

When analyzing HOMA-IR as a categorical variable, a fixed-effects model was utilized to pool data from eight studies (14, 17, 18, 22, 32, 34, 37, 39). The outcomes of the analysis showed that individuals who belonged to the highest HOMA-IR group demonstrated an augmented occurrence of CAC among subjects who fell within the lowest classification [OR: 1.13, 95% CI 1.06–1.20, I^2^ = 29%, P <0.001; **Figure 2A**]. A similar outcome was obtained when HOMA-IR was treated as a continuous variable (15, 23, 32, 33, 35–37). The application of a random-effects model to analyze the data revealed that higher HOMA-IR values were also correlated with an elevated prevalence of CAC [OR: 1.27, 95% CI: 1.14–1.41, I^2^ = 54%, P < 0.001; **Figure 2B**].

Upon conducting sensitivity analyses, we observed that removing any of the studies did not significantly alter heterogeneity or pooled OR values. Specifically, the ORs for categorical variables ranged from 1.11 to 1.18. Likewise, the ORs for continuous variables ranged from 1.24 to 1.30. All of the above analyses demonstrate P values less than 0.001. See **Tables 2** and **3**.

**Table 2:** Results of sensitivity analysis when the HOMA-IR was applied as a categorical variable.

Dataset excluded	OR	95% CI	I^2^%	P for effect
Bertoni 2007 (17) Men	1.18	1.08–1.29	25	<0.001
Bertoni 2007 (17) Women	1.13	1.05–1.22	37	<0.001
Blaha 2011 (18)	1.13	1.06–1.21	34	<0.001
Echouffo-Tcheugui 2019 (32)	1.12	1.06–1.21	31	<0.001
Jorge-Galarza 2016 (34)	1.13	1.05–1.20	35	<0.001
Jung 2015 (22)	1.13	1.06–1.21	36	<0.001
Ke 2023 (38)	1.11	1.04–1.19	0	<0.001
Kim 2017 (39)	1.12	1.04–1.19	10	<0.001
Sung 2013 (37) Men	1.12	1.05–1.20	35	<0.001
Sung 2013 (37) Women	1.13	1.06–1.20	37	<0.001

HOMA-IR, homeostasis model assessment of insulin resistance.

**Table 3:** Results of sensitivity analysis when the HOMA-IR was applied as a continuous variable.

Dataset excluded	OR	95% CI	I^2^%	P for effect
Echouffo-Tcheugui 2019 (32)	1.29	1.15–1.45	55	<0.001
Fakhrzadeh 2016 (33) DM	1.30	1.18–1.44	41	<0.001
Fakhrzadeh 2016 (33) Non-DM	1.25	1.13–1.38	51	<0.001
He 2012 (23)	1.29	1.14–1.46	56	<0.001
Mehta 2011 (35) DM	1.25	1.13–1.39	54	<0.001
Mehta 2011 (35) Non-DM	1.24	1.12–1.38	52	<0.001
Reilly 2004 (36)	1.25	1.12–1.39	53	<0.001
Sung 2013 (37) Men	1.28	1.14–1.45	58	<0.001
Sung 2013 (37) Women	1.28	1.15–1.42	55	<0.001
Yamazoe 2016 (15)	1.27	1.13–1.42	58	<0.001

HOMA-IR, homeostasis model assessment of insulin resistance; DM, diabetes mellitus.

### Subgroup analysis

To account for the potential impact of different clinical characteristics of study subjects on the results, we conducted a subgroup analysis (refer to **Table 4**). Our analysis revealed that the source of study participants did not significantly affect the pooled ORs in either community or non-community residents, physical examination, or non-physical examination population (all P > 0.05).

**Table 4:** Risk of CAC according to different characteristics of included studies.

Subgroup analysis	No. of studies	OR	95% CI	I^2^%	P for subgroup difference
Is a community resident	8	1.13	1.06–1.20	29	0.11
Yes	4	1.10	1.02–1.18	42	
No	4	1.25	1.08–1.45	0	
Whether it is a health checkup population	9	1.13	1.06–1.20	22	0.10
Yes	4	1.25	1.08–1.45	0	
No	5	1.10	1.02–1.18	28	
Included CVD participants or not	7	1.25	1.12–1.39	52	0.39
Excluded	5	1.27	1.09–1.48	61	
Included	2	1.20	1.08–1.32	0	
Participants with DM or not	8	1.27	1.14–1.41	54	0.83
Yes	2	1.22	0.72–2.05	80	
No	8	1.29	1.17–1.42	42	
CAC score	7	1.25	1.12–1.39	52	0.96
Agatston score >0	4	1.26	1.10–1.43	32	
Non-Agatston score >0	3	1.25	1.02–1.53	73	

CAC, coronary artery calcification; CVD, cardiovascular disease; DM, diabetes mellitus.

We conducted a risk factor analysis for CVD. Our analysis included examining whether participants with CVD were excluded from the study population. When HOMA-IR was used as a continuous variable, there was no statistical difference in subgroups between complete exclusion and partial exclusion/no exclusion of participants with CVD (P > 0.05). When HOMA-IR was used as a categorical variable, one study did not exclude patients with CVD from the population (38). However, our sensitivity analysis in **Table 2** showed that excluding this study did not significantly change the results. Considering that IR might differ in diabetes, we performed subgroup analyses according to whether participants had diabetes or not. The results showed that the association between IR and CAC was statistically significant only among the non-diabetic subgroup (OR: 1.29, 95% CI: 1.17–1.42) but not in the diabetes subgroup (OR: 1.22, 95% CI: 0.72–2.05).

Due to data limitations, subgroup analyses for people with or without hypertension and different lipid profiles could not be performed. However, out of all the studies in the multifactorial analysis, only one did not adjust for hypertension/blood pressure (36) and the other did not adjust for lipid status (23). Notably, excluding these two studies from the sensitivity analysis did not affect the overall combined-OR value.

In addition, differences in CAC prevalence evaluation criteria among the included studies were considered. Subgroup analysis showed that an Agatston score >0 did not show a difference from the subgroup with a non-Agatston score > 0.

### HOMA-IR and CAC progression

A meta-analysis of two longitudinal cohort research (14, 31) showed that HOMA-IR was statistically related to CAC progression when used as a categorical variable (OR: 1.44, 95% CI 1.04–2.01, I^2^ = 21%, P < 0.05). In addition, a cohort study (15) of community residents investigating HOMA-IR as a continuous variable identified its independent relationship with CAC progression (OR: 1.25, 95% CI: 1.01–1.55, P < 0.05).

### Publication bias

**Figure 3:**
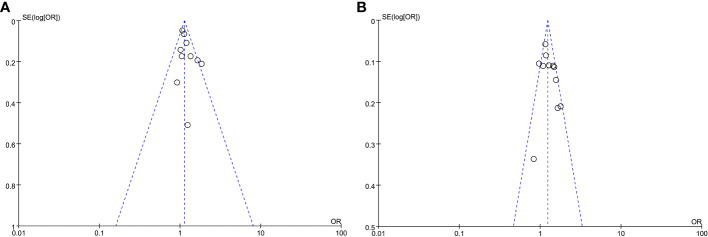
Meta-analysis funnel plots of the HOMA-IR with CAC prevalence. **(A)** HOMA-IR was applied as a categorical variable. **(B)** HOMA-IR was applied as a continuous variable.

In order to evaluate the probability of publication bias, we employed funnel plots. The scatter on the funnel plot displayed a visually balanced distribution, thereby suggesting a reduced likelihood of publication bias (**Figure 3**). Additionally, the outcomes from the Egger regression analysis remained consistent when examining HOMA-IR as both a categorical and continuous variable (P =0.149 and 0.297, respectively).

### Overall body of evidence quality

Of the downgrading factors regarding the CAC prevalence, in terms of study limitations and indirectness, the included studies have a broad representation of the population and use standard measurement methods for both exposure and outcome indicators, which reduces potential concerns. At the same time, the point estimates of the included studies show high similarity, with overlapping confidence intervals in most studies. Additionally, there was low to moderate heterogeneity among the included studies, all of these indicating consistencies. The study’s large sample size also mitigates concerns about imprecision. Furthermore, there is no evidence of publication bias, eliminating any concern in this regard. However, the quality of evidence cannot be upgraded as the pooled OR values are all less than 2. Confounding factors pose a challenge as not all studies excluded patients with cardiovascular disease and only some considered insulin/hyperglycemic drugs. These factors prevent us from eliminating concerns about plausible residual biases. Furthermore, the inability to evaluate the dose–response relationship also hinders its upgrade. Regarding CAC progression, we have concerns about potential bias in outcome measures because of the use of different Agatston method-based evaluation methods in each of the three included studies. Therefore, the quality of evidence rating will directly be very low.

## Discussion

In the present meta-analysis of observational studies, we investigated the correlation between IR and CAC, as evaluated by the HOMA-IR method. The findings revealed a noteworthy correlation between HOMA-IR and the prevalence and progression of CAC. Regarding CAC prevalence, comparable outcomes were observed regardless of whether HOMA-IR was analyzed as a categorical variable or continuous. In relation to CAC progression, the pooled analysis indicated that HOMA-IR was also positively linked with CAC progression when analyzed as a categorical variable. Sensitivity analyses and subgroup analyses based on different clinical characteristics confirmed the stability of these results. The level of evidence for the link of HOMA-IR with CAC prevalence and CAC progression was rated as low and very low, respectively.

CAC is significantly linked to various cardiovascular and cerebrovascular disorders. The degree of calcification serves as a dependable marker for coronary atherosclerosis, and the CAC score provides valuable information that surpasses conventional risk factors for heart disease (40). In asymptomatic populations, the CAC score is even the most effective predictor of cardiovascular incidents (41). It can enhance the risk prediction of individuals with borderline or moderate risks. Qualitative assessment of CAC is essential for the earlier identification of coronary atherosclerosis (42). As per the ACC/AHA guidelines (43), individuals with a CAC score exceeding 100 or who place within the 75th percentile are at heightened risk for unfavorable cardiovascular events and, thus, are advised to undergo statin therapy. On the other hand, subjects with a CAC score of 0 displayed a decreased occurrence of cardiovascular events and a substantially lower mortality rate from the disease (44–46). Therefore, identifying the risk factors associated with CAC and screening out potentially high-risk groups may help facilitate early clinical warning.

Research studies have indicated that surrogate markers of IR, such as the metabolic score for IR and the triglyceride glucose index, are linked with a higher risk of CAC (47–49). In particular, the triglyceride glucose index has attracted considerable attention among researchers. However, a review by Solis et al. reveals that the evidence that upholds the application of the index for assessing IR is of moderate-to-low quality (50). As such, the diagnostic efficacy of these alternative indicators for assessing IR requires further rigorous validation.

The assessment of IR through the HOMA-IR model (8, 51) stands as the prevailing approach within clinical research. Its extensive validation has solidified its reliability in evaluating IR (52). In their study, González-González et al. showcased a strong association between the HOMA-IR and a multitude of health outcomes (53). However, the progression of asymptomatic individuals to severe cardiovascular disease often takes decades. Therefore, investigating whether there is a correlation between HOMA-IR and CAC during this period warrants attention.

As far as we are aware, this meta-analysis is the first examination of the correlation between HOMA-IR and CAC. Our discoveries indicate that HOMA-IR exhibits a positive association with CAC for individuals with elevated HOMA-IR, showcasing a 1.13- and 1.27-fold increased likelihood of developing CAC prevalence. These findings hold regardless of whether HOMA-IR was examined as a categorical or continuous variable. The HOMA-IR method was employed in all included studies to evaluate IR. In contrast, the Agatston method was utilized to assess CAC, which is widely accepted in clinical practice and recognized as the gold standard for calcification (11). Thus, the included studies had good homogeneity in their evaluation methods.

Subgroup and sensitivity analyses were performed to account for potential interference from cardiovascular factors. These analyses were based on whether patients with cardiovascular disease were excluded from the baseline data and whether variables such as hypertension or blood pressure and lipid status were adjusted for in the multifactorial analysis. Subgroup analyses were also conducted based on different sources of the study population, the presence of diabetes, and different coronary artery calcification scoring criteria. Despite differences in these factors, our analyses showed similar and robust results across subgroups, except in the diabetes cohort. It should be noted that in the diabetes subgroup, there were only two studies with 716 people. Li et al.’s survey of 1,516 participants with coronary artery disease showed that IR based on triglyceride-glucose index assessment was closely related to coronary artery disease regardless of whether they had diabetes (54). The limited sample size may have reduced statistical power in the subgroup. Additional research is essential to investigating further the association between IR and CAC among individuals with diabetes.

Among the factors for which the overall quality of the evidence was upgraded, large effect sizes and dose–response relationships were not detected. At the same time, concerns were raised about potential confounders, which included glucose/lipid-lowering medication use and the failure of some of the included studies to adequately consider potential cardiovascular as well as diabetic risk factors, a concern reinforced by inconsistent findings based on diabetic subgroups. Thus, the evidence for an association between HOMA-IR and CAC prevalence was not upgraded.

Within a 5.1-year investigation conducted by Lehmann et al., involving a cohort of 3,281 participants, a significant association was discovered between the CAC progression and onset of coronary and cardiovascular events (55). This study is supported by similar findings reported by Budoff et al., wherein CAC progression was identified as an indicator of future events related to coronary heart disease (56). Therefore, we further analyzed the association between HOMA-IR and CAC progression. Three cohort studies examined the link between HOMA-IR and CAC progression (14, 15, 31), and two of these studies utilized HOMA-IR as a categorical variable. Pooled analysis demonstrated a substantial positive correlation between HOMA-IR and CAC progression. The third study presented HOMA-IR as a continuous variable and identified an independent connection between HOMA-IR and CAC progression similarly. The included studies had different definitions of CAC progression, which raises concerns about the risk of bias and indicates that the quality of the evidence is extremely low. In order to further validate the association between CAC progression and IR, it is necessary to develop more standardized evaluation methods in future research.

This study has the following limitations: Firstly, due to the limited data reported in the included studies, it was not possible to analyze the correlation between HOMA-IR and distinct levels of CAC scores. It may be worthwhile to explore what kind of association would be presented at different levels of CAC scores and whether there is a linear association between the two. Secondly, the majority of the included original research comprised cross-sectional studies (10 out of 15). Given the constraints inherent in observational study designs, establishing a causal relationship between IR and CAC becomes unattainable. It is imperative to conduct additional cohort studies with sizeable sample populations. Thirdly, limited to the disadvantages of observational studies, some unobserved confounding variables are likely to bias the results. Although we considered relevant cardiovascular risk factors as much as possible, there may still be variables that were not considered. Fourth, limitations of the available study data prevented us from analyzing the effect of additional factor states, such as different metabolic states, and the effect of drug use, including antihypertensive, hypoglycemic, and lipid-lowering agents, on the connection between HOMA-IR and CAC. Fifth, there were a number of papers with incomplete data that were excluded from the meta-analysis. Although we contacted these authors, no response was received, which may add to the uncertainty of the pooled results. Finally, the current meta-analysis is not registered and may have a minor bias, but we still followed the steps of the systematic review rigorously to produce the research.

## Conclusions

HOMA-IR is a commonly used method in clinical settings to assess IR. This research examined the relationship between IR and CAC based on the HOMA-IR method. The findings of this research propose a noteworthy connection between increased HOMA-IR values and both the prevalence and progression of CAC with low and very low-quality evidence, respectively. It is essential to note that the majority of the original studies included in this research were cross-sectional. As a result, it is imperative to exercise caution when interpreting these findings, and extensive population-based longitudinal studies are imperative to authenticate and corroborate these outcomes in forthcoming research endeavors.

## Data availability statement

The original contributions presented in the study are included in the article/**Supplementary Material**. Further inquiries can be directed to the corresponding author.

## Author contributions

LL: Methodology, Conceptualization, Formal analysis, Visualization, Writing – original draft. HZ: Methodology, Conceptualization, Formal analysis, Validation, Visualization, Writing – review & editing. YS: Supervision, Conceptualization, Validation, Funding acquisition, Writing – review & editing. YH: Data curation, Methodology, Formal analysis, Visualization, Writing – review & editing. XZ: Data curation, Methodology, Formal analysis, Visualization, Writing – review & editing. DL: Data curation, Formal analysis, Software, Writing – review & editing.
