# Supplementary material for: Association between the homeostasis model assessment of insulin resistance and coronary artery calcification: a meta-analysis of observational studies
Source: Front Endocrinol (Lausanne). 2023 Nov 27;14:1271857. doi: 10.3389/fendo.2023.1271857 (PMC10711676; doi:10.3389/fendo.2023.1271857)
Supplement: Supplementary file 1 [file DataSheet_1.doc]

**Supplementary Data Sheet 1: Search strategies**

**PubMed**

#1 ((((((insulin resistance[MeSH Terms])) OR (insulin resistance[Title/Abstract])) OR (homeosta* model assessment[Title/Abstract])) OR (homeosta* model assessment insulin resistance[Title/Abstract])) OR (HOMA[Title/Abstract])) OR (HOMA-IR[Title/Abstract]) 149,046

#2 ((((((((((coronary arter* calcification[Title/Abstract])) OR (coronary arter* calcium[Title/Abstract])) OR (coronary calcification[Title/Abstract])) OR (coronary calcium[Title/Abstract])) OR (subclinical coronary atherosclerosis[Title/Abstract])) OR (subclinical coronary arter* disease[Title/Abstract])) OR (calcifying coronary arter*[Title/Abstract])) OR (calcified coronary arter*[Title/Abstract])) OR (calcifi* coronary disease[Title/Abstract])) OR (calcifi* coronary artery disease[Title/Abstract]) 25,080

#3 #1 AND #2 497

**Embase**

#1 'insulin resistance'/exp OR 'insulin resistance':ti,ab,kw OR 'homeostasis model assessment'/exp OR 'homeosta* model assessment':ti,ab,kw OR 'homeosta* model assessment insulin resistance':ti,ab,kw OR 'HOMA':ti,ab,kw OR 'HOMA-IR':ti,ab,kw 198,869

#2 'coronary artery calcification'/exp OR 'coronary artery calcium'/exp OR 'coronary artery calcium score'/exp OR 'coronary arter* calcification':ti,ab,kw OR 'coronary arter* calcium':ti,ab,kw OR 'coronary calcification':ti,ab,kw OR 'coronary calcium':ti,ab,kw OR 'subclinical coronary atherosclerosis':ti,ab,kw OR 'subclinical coronary arter* disease':ti,ab,kw OR 'calcifying coronary arter*':ti,ab,kw OR 'calcified coronary arter*':ti,ab,kw OR 'calcifi* coronary disease':ti,ab,kw OR 'calcifi* coronary artery disease':ti,ab,kw 20,730

#3 #1 AND #2 529

**Scopus**

#1 TITLE-ABS-KEY(“insulin resistance” OR “homeosta* model assessment” OR “homeosta* model assessment insulin resistance” OR “HOMA” OR “HOMA-IR”) 171,396

#2 TITLE-ABS-KEY(“coronary arter* calcification” OR “coronary arter* calcium” OR “coronary calcification” OR “coronary calcium” OR “subclinical coronary atherosclerosis” OR “subclinical coronary arter* disease” OR “calcifying coronary arter*” OR “calcified coronary arter*” OR “calcifi* coronary artery disease”) 14,498

#3 #1 AND #2 410

**Web of science**

#1 “insulin resistance” OR “insulin resistance” OR “homeosta* model assessment” OR “homeosta* model assessment insulin resistance” OR “HOMA” OR “HOMA-IR” (topic) 224,896

#2 “coronary arter* calcification” OR “coronary arter* calcium” OR “coronary calcification” OR “coronary calcium” OR “subclinical coronary atherosclerosis” OR “subclinical coronary arter* disease” OR “calcifying coronary arter*” OR “calcified coronary arter*” OR “calcifi* coronary disease” OR “calcifi* coronary artery disease” (topic) 15,384

#1 AND #2 591
